# A study on the application and effect of ERAS-based refined nursing in patients undergoing radical lung cancer surgery

**DOI:** 10.3389/fsurg.2025.1557289

**Published:** 2025-05-07

**Authors:** Fang Qi, Bin Wang, Xiangnan Li, Yan Wang, Jing Zhang

**Affiliations:** Thoracic Surgery Department, Tangshan People’s Hospital of Hebei Province, Tangshan, Hebei, China

**Keywords:** enhanced recovery after surgery, refined nursing care, radical resection of lung cancer, quality of life, survival analysis

## Abstract

**Background:**

In recent years, the Enhanced Recovery After Surgery (ERAS) concept has gradually been applied in clinical practice. The aim of this study is to evaluate the impact of refined nursing based on the ERAS concept on clinical index, physiological index, and comprehensive health index of patients undergoing radical surgery for lung cancer.

**Method:**

This study included 120 patients who underwent radical surgery for lung cancer. All patients were divided into an observation group (receiving refined nursing based on ERAS concept) and a control group (receiving routine care). Analyze the differences in clinical index, physiological index, comprehensive health index, and patient satisfaction at discharge between two groups before and after intervention. Evaluate the independent impact of nursing methods on these indicators through multiple linear regression. The ROC curve is used to evaluate the performance of a multi factor linear regression model.

**Result:**

The observation group showed significant improvement in clinical index, physiological index, and comprehensive health index compared to the control group. In terms of complications, the incidence of pneumonia, pneumothorax and other complications in the observation group was significantly lower than that in the control group. The results of multiple linear regression analysis showed that ERAS based refined nursing had significant independent effects on clinical index, physiological index, and comprehensive health index. The ROC curve shows that the AUC of the biochemical indicator model is the highest, followed by the comprehensive health indicator and clinical indicator models.

**Conclusion:**

The refined nursing based on ERAS concept significantly improves the clinical index, physiological index, and comprehensive health index of lung cancer radical surgery patients compared to conventional nursing, and has the best effect in physiological index.

## 1 Introduction

Lung cancer is one of the most common malignant tumors, with the highest mortality rate and the second highest incidence rate (1–3). The patient mainly presents with coughing up blood, chest pain, and long-term cough, which seriously affects their life and quality of life, and has a low survival rate within 5 years (4, 5). Currently, surgical treatment is the preferred treatment for lung cancer patients in clinical practice (6, 7). With the continuous advancement of medical technology, thoracoscopic guided radical resection of lung cancer has been widely used (8). However, research has found that there is a high demand for nursing cooperation during the treatment process, and scientific nursing cooperation needs to be implemented at the appropriate time (9).

Refined nursing is a patient-centered nursing service concept that advocates proactive, humanized, and emotional services (10). It can improve patients' self-awareness, confidence, self-efficacy, and treatment compliance, and has a certain auxiliary effect on their recovery. Nursing staff, hospitals, departments, etc. can all improve the service capabilities of their clients, form a good nurse patient relationship, and improve the medical environment. Previous studies have shown that the application of refined nursing interventions in various diseases can effectively improve the quality of life of patients (11). Postoperative pain in lung cancer patients can cause significant damage, and refined nursing can improve patients' understanding of the disease and their awareness of unhealthy lifestyle habits, which can promote disease recovery. Enhanced recovery after surgery (ERAS), which emerged in the early 21st century, is a new nursing model guided by evidence-based medicine to optimize nursing plans, reduce postoperative trauma and complications, lower the incidence of stress reactions, improve prognosis, and accelerate recovery (12, 13). In recent years, this nursing model has gradually been applied to the care of different cancer patients and has achieved certain results.

The integration of ERAS with refined nursing involves implementing the ERAS concept throughout the refined nursing process before, during, and after surgery. For example, one-on-one health education is adopted before surgery. The operating room temperature is adjusted 30 min before surgery, and the patient's temperature changes are monitored and adjusted in a timely manner during surgery. After surgery, activities such as back percussion, repositioning, coughing, and expectoration are combined with lung function exercise to promote early recovery of the patient. Attention is also paid to pain management and emotional changes, and psychological counseling and care are provided in a timely manner. These are all in line with the ERAS philosophy of reducing physiological and psychological stress, optimizing temperature management, and promoting early postoperative activity.

At present, there have been studies on the application of ERAS in lung cancer, but these studies are limited to meta-analysis and reviews (14, 15). Even if there are clinical studies, their outcome variables are not comprehensive enough, mainly including complications and some surgical related indicators (16, 17). Refined nursing is also widely used in research on lung cancer and other cancers (such as colon cancer). The results show that refined nursing can effectively improve the quality and efficiency of surgical care, and increase patient satisfaction with nursing staff (18, 19), but there are few studies that combine ERAS concepts with refined nursing. Compared with previous studies, this study added blood gas indicators, psychological status indicators, and quality of life indicators and conducted multiple linear regression analysis. The ERAS concept combined with refined nursing not only has novel and unique characteristics, but also provides a more comprehensive, systematic, and detailed nursing strategy. Evaluating the application of precision nursing based on ERAS concept in lung cancer radical surgery patients can help improve clinical efficacy, stabilize patient condition, improve patient psychological state, reduce the occurrence of complications, and comprehensively improve nursing quality.

## 2 Materials and methods

### 2.1 Research object

120 lung cancer patients who underwent radical surgery for lung cancer in our hospital from January 2022 to January 2024 were selected as the research subjects, and were divided into an observation group (accelerated rehabilitation surgery concept combined with refined nursing intervention) and a control group (conventional nursing intervention) according to different nursing methods. Inclusion criteria: Meets the diagnostic criteria for lung cancer in the Clinical Diagnosis and Treatment Guidelines for Lung Cancer of the Chinese Medical Association (2018 Edition), and is diagnosed with lung cancer through pathological and imaging examinations; Age >22 years old; Accept radical surgery for lung cancer; All clinical data is complete. Exclusion criteria: History of mental illness; Severe functional impairment of important organs such as the heart, liver, and kidneys; Combined coagulation system diseases; Combine severe lung diseases such as asthma, respiratory malformations, and chronic obstructive pulmonary disease. We used a random number table to assign patients to different groups. To ensure the randomness of allocation and avoid potential bias, sealed envelopes are used to keep the allocation concealment, thereby minimizing selection bias to the greatest extent.

### 2.2 Nursing methods

Control group: Implement routine care until the patient is discharged. Before surgery, nursing staff provide oral education to patients on disease knowledge, surgical procedures, and precautions, and prepare for preoperative work. During the operation, perform routine operating room care and actively cooperate with the physician to complete the surgery. After surgery, real-time electrocardiogram monitoring is performed on patients, and routine antibiotic treatment and analgesic treatment are carried out according to medical advice. Patients are encouraged to undergo appropriate rehabilitation training within their physical tolerance range, and scientific dietary management and life guidance are provided.

Observation group: Implement the concept of accelerated rehabilitation surgery combined with refined nursing intervention on the basis of the control group until the patient is discharged. The main content includes preoperative refined nursing: establishing electronic records for patients and conducting detailed evaluations, including medical history, allergy history, and surgical history. Through one-on-one health education, introduce patients and their families to diseases, surgical safety, and postoperative precautions, and share successful cases to alleviate patient anxiety. Advise patients to fast for 6 h before surgery and to abstain from water for 4 h before surgery. Fine care during surgery: Adjust the operating room temperature 30 min before surgery to ensure a warm environment. Verify patient information and provide detailed information during transportation to ensure patient comfort. Closely monitor body temperature during surgery and adjust the temperature of the environment and surgical supplies to ensure appropriate fluid temperature, and implement restricted fluid replacement. Postoperative refined nursing: Postoperative nurses assist patients with back tapping and turning, every 1–2 h, to maintain airway patency. After the patient wakes up, tilt their head to one side to avoid aspiration of secretions. Guide the patient to cough and expectorate within 2 days after surgery, and use a suction device if necessary. Postoperative dietary guidance will be provided by a nutritionist. After the patient's intestinal function recovers, they start a semi liquid or liquid diet and gradually transition to a normal diet. Start lung function training after surgery, first perform pursed lip breathing training, lasting 3–5 min each time, 3–5 times a day; Then perform balloon blowing training to help increase lung capacity. Strengthen daily ward rounds and visits, pay attention to patients' emotional changes, and provide psychological support. Pay attention to both incision pain and recovery, and explain the cause of pain in a timely manner.

### 2.3 Data collection

Collect patients' age, gender, BMI, Smoking status, drinking status, preoperative nutrition status, clinical stage, cardiovascular disease, diabetes, kidney disease, liver disease, compare the operation time, tracheal extubation time, time to first flatus, drainage tube retention time, autonomous cough time, out of bed activity time and hospitalization time of each group. Visual Analog Scale (VAS) was used to assess pain scores for two groups of patients upon admission and at 24, 48, and 72 h postoperatively. Compare the changes in lung function indicators between two groups of patients at admission and 3 days after surgery, including forced expiratory volume in one second (FEV1), forced vtal capacity (FVC), and peak expiratory flow (PEF) day night variability. Compare the blood gas indicators of two groups of patients at admission and 3 days after surgery, including Pulse Oximetry Saturation (SpO2), Arterial Oxygen Partial Pressure (PaO2), and Arterial Carbon Dioxide Partial Pressure (PaCO2). Record and compare the incidence of postoperative complications within one year between the two groups, including pulmonary atelectasis, pneumonia, pneumothorax, etc.

Two groups of patients were evaluated for their psychological status using the Hamilton Depression Rating Scale (HAMD) and the Hamilton Anxiety Rating Scale (HAMA) at admission and 3 days after surgery. The Quality of Life Scale (QLQ-30) was used to compare the quality of life of two groups of patients at admission and 3 days after surgery. Compare the disease cognition level and self-management ability of two groups of patients at admission and before discharge using the Brief Illness Perception Questionnaire (B-IPQ) and the Self-Reported Use of Patient-Health Promotion (SUPPH). Use the Newcastle Satisfaction with Nursing Scale (NSNS) to assess patient satisfaction with nursing services. The maximum score is 95 points, with 57 points or above indicating satisfaction, and the following indicating dissatisfaction.

Pulmonary atelectasis is usually diagnosed through imaging examinations (chest x-rays or CT scans), characterized by lung parenchyma collapse or loss of aeration in lung regions or lobes, leading to inadequate ventilation. Pneumonia is characterized by pulmonary infiltrates on chest x-rays or CT scans in the lungs on chest x-rays or CT scans. Pneumothorax is characterized by chest x-rays or CT scans showing gas in the chest cavity and lung collapse. DVT is primarily diagnosed using Doppler ultrasound of the lower extremities. Pulmonary embolism is confirmed by computed tomography pulmonary angiography (CTPA). Postoperative bleeding is confirmed by clinical symptoms such as decreased blood pressure, pale skin, increased heart rate, and bleeding points. Arrhythmia is usually diagnosed through electrocardiography (ECG).

Among them, we classified surgical time, extubation time, time to first flatus, drainage tube retention time, time to spontaneous cough, time to first ambulation, and length of hospital stay as clinical index. The VAS pain score, forced expiratory volume in 1 s (FEV1), forced vtal capacity (FVC), peak expiratory flow (PEF) day night variability, Pulse Oximetry Saturation (SpO2), Arterial Oxygen Partial Pressure (PaO2), and Arterial Carbon Dioxide Partial Pressure (PaCO2) were classified as physiological index. Classify the scores of Hamilton Depression Rating Scale (HAMD), Hamilton Anxiety Rating Scale (HAMA), Quality of Life Scale (QLQ-30), Brief Illness Perception Questionnaire (B-IPQ), and Self-Reported Use of Patient-Health Promotion (SUPPH) as comprehensive health index.

### 2.4 Statistical analysis

Use R 4.4.1 software for data analysis. Categorical variables are expressed in frequency (percentage) using chi square test or Fisher's exact test, while continuous variables are expressed in median (minimum-maximum) using independent samples *t*-test or Mann–Whitney *U* test. Standardize the *Z*-score of clinical indicators, biochemical indicators, and comprehensive health indicators for each factor within 3 days after surgery, and then add them together. Negative values should be taken for negative factors in biochemical indicators and comprehensive health indicators (such as HAMD, HAMA). We obtained the total scores of clinical indicators, biochemical indicators, and comprehensive health indicators separately, and used these three total scores as dependent variables for multiple regression analysis to evaluate the independent effects of nursing methods on clinical indicators, biochemical indicators, and comprehensive health indicators. The ROC curve is used to evaluate the performance of multiple regression models.

## 3 Results

### 3.1 Demographic characteristics of control group and observation group

The age range of all 120 lung cancer radical surgery patients was 38–69 years (median 52 years). Among them, there were 67 males (55.83%) and 53 females (44.17%). The BMI range is 17.26–36.86 (median 26.65). Among smokers, 55% have never smoked, 28.33% have smoked, and 16.67% are current smokers. The drinking situation shows that 50.83% have never consumed alcohol, 19.17% have consumed alcohol, and 30% are current drinkers. In terms of preoperative nutritional status, 74.17% were well nourished and 25.83% were malnourished. In clinical staging, 62.5% are in stage I, 30% are in stage II, and 7.5% are in stage III. As for chronic diseases, 11.67% of patients have cardiovascular diseases, 10% have diabetes, 6.67% have kidney diseases, and 4.17% have liver diseases. 74.17% of the patients underwent lobectomy, 20% underwent segmentectomy/sub-lobectomy, and 5.83% underwent wedge resection. Additionally, 73.33% of the patients opted for video-assisted thoracic surgery (VATS), while 26.67% chose thoracotomy. The proportion of male patients in the observation group was significantly lower than that in the control group in terms of cardiovascular disease, and their smoking habits were also significantly different from those in the control group. There were no significant differences in other indicators between the two groups. Overall, the majority of patients are in good health condition, and the distribution of the two groups of patients is relatively balanced (Table 1).

**Table 1:** Demographic characteristics of lung cancer patients in the control group and the observation group.

Variable	All patients (*n* = 120)	Control group (*n* = 60)	Observation group (*n* = 60)	*P-*value
Age	52 (38–69)	52 (38–68)	52 (38–69)	0.459
Gender				0.0274
Male	67 (55.83%)	40 (66.67%)	27 (45%)	
Female	53 (44.17%)	20 (33.33%)	33 (55%)	
BMI	26.65 (17.26–36.86)	27.25 (17.53–36.65)	26.32 (17.26–36.86)	0.725
Smoking				0.014
Never smoked	66 (55%)	29 (48.33%)	37 (61.67%)	
Former smoker	34 (28.33%)	24 (40%)	10 (16.67%)	
Current smoker	20 (16.67%)	7 (11.67%)	13 (21.67%)	
Drinking				0.0968
Never drank alcohol	61 (50.83%)	32 (53.33%)	29 (48.33%)	
Former drinker	23 (19.17%)	7 (11.67%)	16 (26.67%)	
Current drinker	36 (30%)	21 (35%)	15 (25%)	
Preoperative nutritional status				0.0952
Well-nourished	89 (74.17%)	40 (66.67%)	49 (81.67%)	
Malnourished	31 (25.83%)	20 (33.33%)	11 (18.33%)	
Clinical stage				0.0526
Stage I	75 (62.5%)	40 (66.67%)	35 (58.33%)	
Stage II	36 (30%)	19 (31.67%)	17 (28.33%)	
Stage III	9 (7.5%)	1 (1.67%)	8 (13.33%)	
Cardiovascular diseases				0.0465
Yes	14 (11.67%)	11 (18.33%)	3 (5%)	
No	106 (88.33%)	49 (81.67%)	57 (95%)	
Diabetes mellitus				0.1281
Yes	12 (10%)	9 (15%)	3 (5%)	
No	108 (90%)	51 (85%)	57 (95%)	
Renal diseases				0.0673
Yes	8 (6.67%)	7 (11.67%)	1 (1.67%)	
No	112 (93.33%)	53 (88.33%)	59 (98.33%)	
Liver diseases				0.3609
Yes	5 (4.17%)	1 (1.67%)	4 (6.67%)	
No	115 (95.83%)	59 (98.33%)	56 (93.33%)	
Surgical types				0.1467
Lobectomy	89 (74.17%)	46 (76.67%)	43 (71.67%)	
Segmentectomy/sub-lobectomy	24 (20%)	13 (21.67%)	11 (18.33%)	
Wedge resection	7 (5.83%)	1 (1.67%)	6 (10%)	
Surgical approach				0.1485
Video-assisted thoracic surgery, VATS	88 (73.33%)	40 (66.67%)	48 (80%)	
Thoracotomy	32 (26.67%)	20 (33.33%)	12 (20%)	

### 3.2 Differences in multiple clinical index between the control group and the observation group of lung cancer patients undergoing radical surgery

The observation group had significantly lower surgical time (*P* = 0.0209), extubation time (*P* = 0.00364), time to first flatus (*P* = 0.0322), drainage tube retention time (*P* = 0.0353), spontaneous cough time (*P* = 0.0162), first mobilization time (*P* = 0.045), and hospitalization time (*P* = 0.0145) compared to the control group, indicating that the recovery process of the observation group was relatively faster, the hospitalization time was shorter, and the overall recovery was smoother than that of the control group (Table 2).

**Table 2:** Clinical indicators differences between the control group and the observation group.

Variable	All Patients (*n* = 120)	Control group (*n* = 60)	Observation group (*n* = 60)	*P*-value
Surgical Time (min)	244 (126–341)	279 (126–340)	226 (129–341)	0.0209
Extubation time (h)	8.5 (6.3–11.5)	8.8 (6.4–11.5)	8.3 (6.3–11.3)	0.00364
Time to First Flatus (h)	22.9 (10.6–30.3)	24.3 (10.9–30.0)	22.1 (10.6–30.3)	0.0322
Drainage Tube Retention Time (day)	3.4 (2.2–4.5)	3.6 (2.2–4.5)	3.3 (2.3–4.5)	0.0353
Time to Spontaneous Cough (h)	25.9 (16.4–35.0)	28.3 (17.0–35.0)	25.3 (16.4–35.0)	0.0162
Time to First Ambulation (h)	36.0 (26.3–45.7)	36.7 (26.5–45.7)	35.0 (26.3–45.1)	0.045
Length of Hospital Stay (day)	7.0 (5.4–9.3)	7.4 (5.4–9.3)	6.7 (5.4–9.0)	0.0145

### 3.3 Differences in biochemical and comprehensive health index between the observation group and the control group before and after intervention

In terms of physiological index, there was no significant difference in preoperative pain scores between the two groups. 24 h after surgery, the pain scores of the observation group were significantly lower than those of the control group. At 48 and 72 h after surgery, the pain scores of the observation group were significantly lower than those of the control group, indicating that the pain relief in the observation group was faster. Before surgery, there was no significant difference in the diurnal fluctuations of FEV1, FVC, and PEF between the two groups. After surgery, the FEV1, FVC, and PEF fluctuations in the observation group were significantly lower than those in the control group. Before surgery, there was no significant difference in SpO2, PaO2, and PaCO2 between the two groups. After surgery, the SpO2 and PaO2 in the observation group were significantly higher than those in the control group, while PaCO2 was significantly lower than those in the control group. Before surgery, there was no significant difference in HAMD and HAMA scores between the two groups. After surgery, the HAMD and HAMA scores of the observation group were significantly lower than those of the control group. Before surgery, there was no significant difference in QLQ-30, B-IPQ, and SUPPH scores between the two groups. After surgery, the QLQ-30, B-IPQ, and SUPPH scores in the observation group were significantly higher than those in the control group (Table 3).

**Table 3:** Differences in biochemical indicators and overall health indicators between the observation group and the control group.

Variable	All Patients (*n* = 120)	Control group (*n* = 60)	Observation group (*n* = 60)	*P*-value
Physiological index				
Pain score				
Admission	6 (5–8)	6 (5–8)	6 (5–8)	0.954
24 h after surgery	8 (6–9)	8 (6–9)	7 (6–9)	0.049
48 h after surgery	6 (5–7)	6 (5–7)	6 (5–7)	0.0417
72 h after surgery	5 (3–6)	5 (3–6)	4 (3–6)	0.0151
Forced expiratory volume in 1 s (FEV1) diurnal variation rate (%)				
Admission	10.8 (7.1–15.0)	10.5 (7.1–15.0)	11.0 (7.1–15.0)	0.0521
Three days after surgery	18.7 (13.4–22.9)	19.3 (13.6–22.9)	17.8 (13.4–22.6)	0.0221
Forced vital capacity (FVC) diurnal variation rate (%)				
Admission	9.3 (5.8–12.6)	8.8 (6.0–12.5)	9.5 (5.8–12.6)	0.769
Three days after surgery	17.6 (14.7–21.6)	18.0 (14.8–21.4)	16.7 (14.7–21.6)	0.0322
Peak expiratory flow (PEF) diurnal variation rate (%)				
Admission	8.5 (5.4–11.6)	8.5 (5.4–11.5)	8.6 (5.5–11.6)	0.896
Three days after surgery	16.1 (12.7–19.2)	16.9 (13.1–19.2)	15.4 (12.7–19.1)	0.00498
Pulse oximetry saturation (SpO2)				
Admission	98.0 (96.2–99.8)	98.0 (96.2–99.7)	98.1 (96.3–99.8)	0.733
Three days after surgery	92.7 (89.1–96.3)	91.8 (89.1–96.2)	93.7 (89.2–96.3)	0.00605
Arterial oxygen partial pressure (PaO2)				
Admission	84.2 (70.5–95.0)	84.8 (70.5–94.2)	83.2 (71.5–95.0)	0.896
Three days after surgery	68.7 (63.2–74.5)	67.1 (63.2–73.9)	71.1 (63.7–74.5)	0.00263
Arterial carbon dioxide partial pressure (PaCO2)				
Admission	43.4 (38.4–47.0)	43.5 (38.5–47.0)	43.3 (38.4–47.0)	0.574
Three days after surgery	45.8 (41.3–49.6)	46.7 (41.3–49.6)	45.3 (41.8–49.6)	0.00909
Comprehensive health index				
HAMD				
Admission	7 (4–9)	7 (4–9)	6 (4–9)	0.255
Three days after surgery	11 (7–14)	12 (7–14)	10 (7–14)	0.00098
HAMA				
Admission	10 (6–13)	10 (6–13)	10 (6–13)	0.582
Three days after surgery	18 (15–21)	19 (15–21)	17 (15–21)	3.15E−06
QLQ-30				
Admission	56 (45–68)	56 (45–68)	57 (45–67)	0.879
Three days after surgery	61 (49–71)	58 (49–70)	64 (49–71)	9.42E−05
B-IPQ				
Admission	45 (39–52)	45 (39–52)	46 (39–52)	0.607
Three days after surgery	64 (58–70)	62 (58–70)	66 (58–70)	0.013
SUPPH				
Admission	89 (75–104)	87 (76–104)	90 (75–104)	0.614
Three days after surgery	108 (96–118)	104 (96–118)	110 (96–117)	0.0331

### 3.4 Differences in complications between the observation group and the control group

In terms of atelectasis, the incidence rate was 14.17% in all patients, 8.33% in the control group, and 20% in the observation group, with no statistically significant difference (*P* = 0.116). In terms of pneumonia, the overall incidence rate was 10%, with a higher incidence rate in the control group (18.33%) and a lower incidence rate in the observation group (1.67%), and the difference was statistically significant (*P* = 0.006). The incidence of pneumothorax was 7.5%, 13.33% in the control group, and 1.67% in the observation group, with a statistically significant difference (*P* = 0.038). The incidence of deep vein thrombosis/pulmonary embolism was 13.33%, with a higher incidence in the control group (23.33%) and a lower incidence in the observation group (3.33%), and the difference was statistically significant (*P* = 0.003). The incidence of postoperative bleeding was 5.83%, with a higher incidence in the control group (11.67%) and no occurrence in the observation group (*P* = 0.019). Finally, the incidence of arrhythmia was 12.5%, higher in the control group (18.33%) and lower in the observation group (6.67%), but the difference was not statistically significant (*P* = 0.098) (Table 4).

**Table 4:** Difference in complications between the observation group and the control group.

Variable	All Patients (*n* = 120)	Control group (*n* = 60)	Observation group (*n* = 60)	*P*-value
Atelectasis				0.11625
Yes	17 (14.17%)	5 (8.33%)	12 (20%)	
No	103 (85.83%)	55 (91.67%)	48 (80%)	
Pneumonia				0.00617
Yes	12 (10%)	11 (18.33%)	1 (1.67%)	
No	108 (90%)	49 (81.67%)	59 (98.33%)	
Pneumothorax				0.03757
Yes	9 (7.5%)	8 (13.33%)	1 (1.67%)	
No	111 (92.5%)	52 (86.67%)	59 (98.33%)	
Deep vein thrombosis/pulmonary embolism				0.00314
Yes	16 (13.33%)	14 (23.33%)	2 (3.33%)	
No	104 (86.67%)	46 (76.67%)	58 (96.67%)	
Postoperative bleeding				0.01944
Yes	7 (5.83%)	7 (11.67%)	0 (0%)	
No	113 (94.17%)	53 (88.33%)	60 (100%)	
Arrhythmias				0.09769
Yes	15 (12.5%)	11 (18.33%)	4 (6.67%)	
No	105 (87.5%)	49 (81.67%)	56 (93.33%)	

### 3.5 Multiple linear regression analysis of the independent impact of nursing methods on perioperative indicators

**Figure 1:**
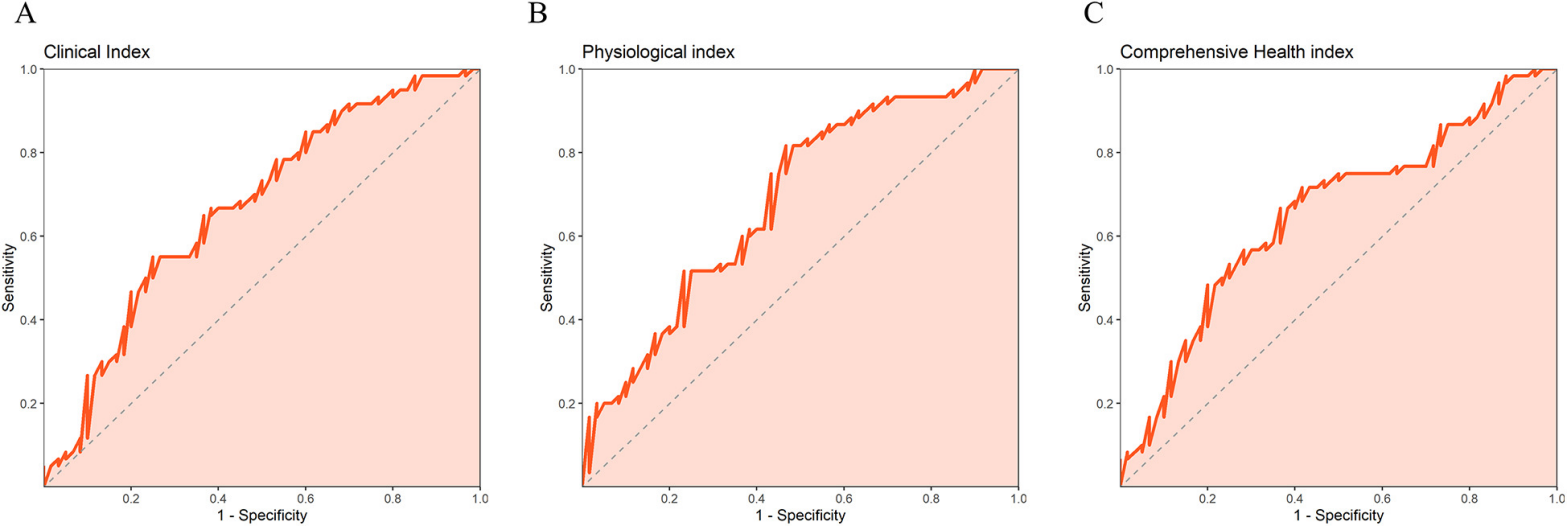
ROC curve of a multiple linear regression model with dependent variables of **(A)** clinical index, **(B)** physiological index, and **(C)** comprehensive health index.

Since gender, smoking history, and cardiovascular disease showed significant differences between the observation group and the control group in the baseline data analysis, while other factors such as age and BMI did not show significant differences, we included these three variables along with the intervention method as independent variables in the multiple linear regression analysis to eliminate the influence of confounding factors. This approach allows us to more accurately assess the independent impact of our intervention method on clinical indicators, biochemical indicators, and overall health outcomes. The results of multiple linear regression showed that in clinical index, the B value of the observation group was −1.251 (*P* = 0.001), indicating that the observation group had significantly lower hospital stay and surgery time than the control group. For physiological index, the B value of the observation group was 1.397 (*P* = 0.004), indicating that the physiological health status of the observation group was significantly better than that of the control group. The B value of the comprehensive health index is 1.158 (*P* = 0.014), which also indicates that the comprehensive health status of the observation group is better than that of the control group. After controlling for gender, smoking status, and potential confounding factors of underlying diseases, nursing methods still showed significant effects on these indicators, indicating that they played an independent role in promoting patient recovery (Table 5). Overall, refined nursing based on the ERAS concept can significantly improve clinical index, physiological index, and comprehensive health index of lung cancer patients compared to conventional nursing, and has the strongest impact on physiological index. The ROC curve also indicates that the AUC of the multiple linear regression model with physiological index as the dependent variable is the highest, followed by clinical index and comprehensive health index (Table 6, Figures 1A–C).

**Table 5:** Multivariate logistic regression was used to analyze the impact of the observation group on the clinical Index, physiological index, and comprehensive health Index.

Variable	Estimate	Std error	Statistic	*P*-value
Clinical index				
Gender (Male vs. Female)	0.629	0.487	1.292	0.197
Smoking (Former smoker vs. Current smoker)	−0.040	0.643	−0.062	0.950
Smoking (Never smoked vs. Current smoker)	−0.922	0.585	−1.540	0.123
Cardiovascular diseases (Yes or No)	0.774	0.657	1.576	0.115
Method (Observation group vs. Control group)	−1.251	0.371	−3.372	0.001
Physiological index				
Gender (Male vs. Female)	−0.062	0.825	−0.075	0.940
Smoking (Former smoker vs. Current smoker)	0.760	0.561	1.355	0.176
Smoking (Never smoked vs. Current smoker)	0.619	0.843	0.734	0.463
Cardiovascular diseases (Yes or No)	−0.922	0.621	−1.485	0.138
Method (Observation group vs. Control group)	1.397	0.484	2.886	0.004
Comprehensive health index				
Gender (Male vs. Female)	−0.276	0.938	−0.294	0.769
Smoking (Former smoker vs. Current smoker)	0.090	0.162	0.556	0.579
Smoking (Never smoked vs. Current smoker)	0.392	0.921	0.426	0.670
Cardiovascular diseases (Yes or No)	0.771	0.476	1.620	0.105
Method (Observation group vs. Control group)	1.158	0.469	2.469	0.014

**Table 6:** ROC curve parameters of the multiple linear regression model.

Index	AUC	AUC-CI-lower	AUC-CI-upper	Best-threshold	Youden	Sensitivity	Specificity
Clinical index	0.668	0.571	0.765	0.413	0.300	0.550	0.750
Physiological index	0.686	0.590	0.781	0.568	0.350	0.817	0.533
Comprehensive health index	0.654	0.555	0.753	0.657	0.300	0.717	0.583

### 3.6 Patient satisfaction difference between the observation group and the control group

**Figure 2:**
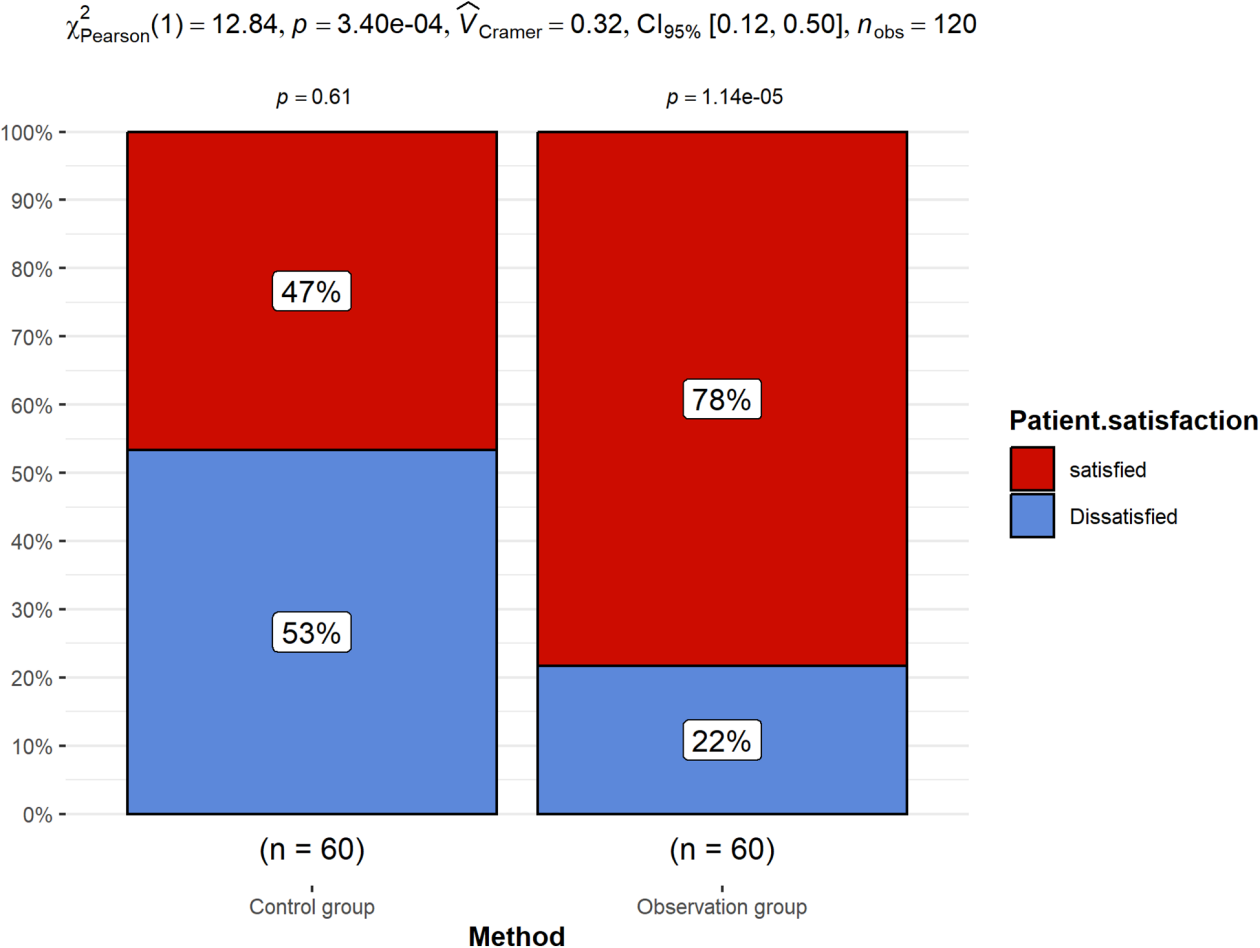
Patient satisfaction between the observation group and the control group.

The satisfaction analysis of nursing services at discharge showed that the satisfaction of the observation group was significantly higher than that of the control group (Figure 2). Refined nursing based on the ERAS concept can effectively enhance patients' nursing experience, increase their recognition and satisfaction with nursing services.

### 3.7 Survival analysis

**Figure 3:**
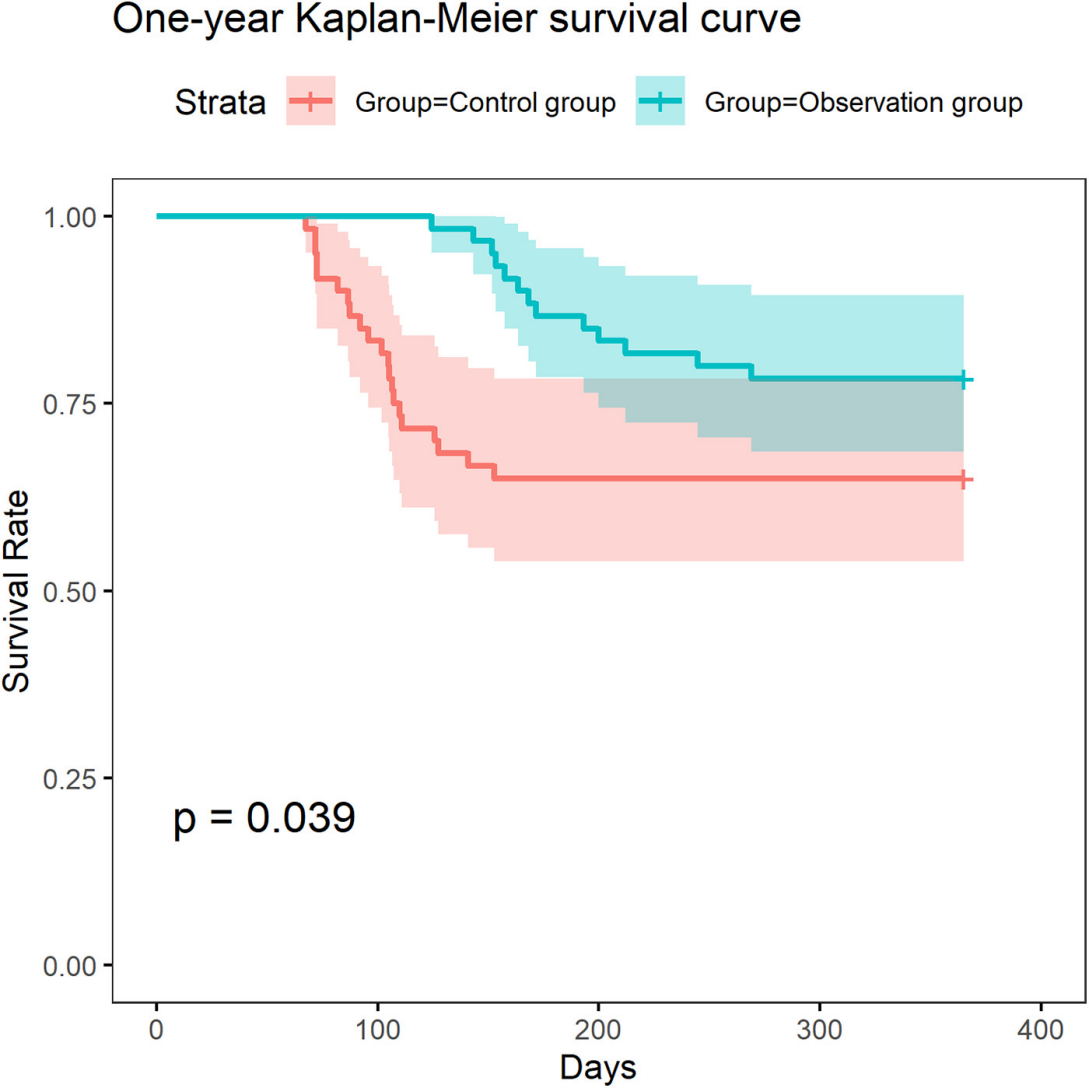
Difference in one-year survival rate between the observation group and the control group.

We conducted a survival analysis based on the survival status of patients during one-year follow-up. The results showed that the survival rate of patients in the observation group was significantly higher than that of the control group (Figure 3). This result indicates that ERAS-based refined nursing can significantly improve the one-year survival outcomes of patients.

## 4 Discussion

This study explores the impact of refined nursing based on the ERAS concept on clinical index, physiological index, and comprehensive health index of patients undergoing radical surgery for lung cancer. The research results showed that the observation group was significantly better than the control group in postoperative recovery, clinical index, pain relief, physiological index, quality of life indicators, psychological status indicators, and incidence of complications, further verifying the role of ERAS concept in postoperative nursing of lung cancer radical surgery.

The observation group showed significantly lower clinical recovery indicators such as surgery duration, extubation time, time to first flatus, and drainage tube retention time compared to the control group, indicating that refined nursing based on the ERAS concept can help shorten postoperative recovery time and reduce postoperative discomfort (20, 21). Refined nursing, through comprehensive interventions before, during, and after surgery, improves patient comfort, reduces postoperative physiological burden, and enables patients to spontaneously cough and get out of bed earlier, thereby accelerating postoperative recovery.

In terms of physiological index, the observation group showed significantly better postoperative pain scores, lung function indicators (FEV1, FVC, PEF fluctuation rate), and blood gas indicators (SpO2, PaO2, PaCO2) compared to the control group. The observation group showed faster pain relief, better recovery of lung function and blood gas indicators, which is closely related to ERAS nursing's preoperative health education, guidance on fasting and water restriction, intraoperative environmental regulation and temperature monitoring, information verification and transportation management, postoperative back tapping and turning, cough and sputum guidance and suction, and lung function training (such as lip tightening breathing training and balloon blowing training). The significant differences in pain scores at 24, 48, and 72 h after surgery indicate the advantages of refined nursing in pain control and comfort, which may be due to personalized care and pain management performed in the early postoperative period (22).

In terms of comprehensive health index, the observation group showed significantly higher HAMD, HAMA scores, QLQ-30, B-IPQ, and SUPPH scores than the control group, indicating that refined nursing not only improved physical health, but also effectively improved patients' psychological state and quality of life. Based on the ERAS concept, refined nursing provides preoperative health education, psychological counseling, and postoperative psychological support to patients, helping to alleviate their anxiety and depression, enhance their confidence in treatment, and ultimately improve their overall health status.

The proportion of patients with preoperative malnutrition in this study was 25.83%, and malnutrition is closely related to the occurrence of postoperative complications. This may be because malnutrition can lead to impaired function of immune cells (such as T cells, B cells, macrophages), thereby reducing immunity and affecting the body's resistance (23). Malnutrition can also reduce wound healing ability, as patients lack protein, which affects collagen synthesis and leads to delayed wound healing after surgery. A considerable proportion (26.67%) of patients opt for open chest surgery, which has a higher incidence of complications compared to thoracoscopy. This may be due to the larger trauma range and longer operation time of open chest surgery, which requires prolonged traction of blood vessels and lung tissue, resulting in greater damage to intercostal nerves, muscles, and ribs. And the pain level after open chest surgery is higher, which affects the patient's deep breathing and effective sputum production, leading to alveolar collapse and increasing the incidence of atelectasis. After open chest surgery, mobility is more restricted, and long-term bed rest can cause venous blood flow to stagnate, thereby increasing the risk of deep vein thrombosis (DVT).

The refined nursing based on ERAS significantly improved the one-year survival rate of patients, indicating that these interventions are not only effective for short-term indicators, but also improve the overall health status and immune function of patients, which is of great significance for their long-term survival and health. This means that ERAS based refined nursing, such as personalized nutritional support, pain management, psychological support, etc., has strong clinical application value.

During the recovery process after radical surgery for lung cancer, patients often face various clinical challenges, such as persistent postoperative pain and its associated complications (such as breathing difficulties and infections). Based on the ERAS concept of refined nursing, early assessment of pain and implementation of personalized pain relief plans have significantly improved their ability to relieve pain compared to conventional nursing, helping to reduce postoperative pain, lower the incidence of complications, and accelerate patient recovery. Secondly, lung cancer patients are prone to postoperative pulmonary complications such as atelectasis and bronchopleural fistula (24, 25), which have a significant impact on their postoperative quality of life. atelectasis is commonly seen in the early postoperative period and is closely related to the recovery of lung function in patients; Bronchopleural fistula is often accompanied by severe respiratory distress and infection, increasing the difficulty of treatment. The personalized methods of postoperative early respiratory training, position guidance, and nutritional support based on the ERAS concept in refined nursing can help enhance patients' lung function and physical fitness, thereby reducing the incidence of complications. In addition, the presence of long-term thoracic drainage may also cause delayed recovery for patients. Scientific drainage management and nursing interventions based on ERAS refined nursing can effectively shorten the time of thoracic drainage, help patients recover normal chest activities as soon as possible, and reduce rehabilitation delays caused by excessive drainage.

Although this study has achieved positive results in patients undergoing radical surgery for lung cancer, there are also certain limitations. Mainly due to the relatively small sample size of this study; In addition, the study only focused on short-term postoperative outcomes and did not delve into the effects in long-term follow-up. And our team lacks the role of a nutritionist, failing to fully consider the role of nutritionists in patient rehabilitation (26). Therefore, future research can further expand the sample size and conduct multi center, long-term follow-up studies, and include nutritionists in the research team to more comprehensively evaluate the long-term impact of the combination of ERAS concept and refined nursing on lung cancer radical surgery patients.

## 5 Conclusion

Overall, refined nursing interventions based on the concept of accelerated rehabilitation surgery have significantly improved various indicators of lung cancer patients undergoing radical surgery. Through meticulous preoperative, intraoperative, and postoperative care, the overall recovery status of patients has been effectively improved, the incidence of postoperative complications has been reduced, and the quality of life of patients has been significantly improved. This provides new ideas and methods for nursing interventions for patients undergoing radical surgery for lung cancer, and has broad clinical application value.

## Data Availability

The original contributions presented in the study are included in the article/Supplementary Material, further inquiries can be directed to the corresponding author.
